# The Modified Gil-Vernet Antireflux Surgery: A Successful Technique for High-Grade Vesicoureteral Reflux Correction in Children—Long-Term Follow-Up

**DOI:** 10.1155/2018/4948165

**Published:** 2018-10-21

**Authors:** Mahmoudreza Moradi, Abolhassan Seyedzadeh, Saeed Gharakhloo, Aref Teymourinezhad, Kaveh Kaseb, Haress Rezaee

**Affiliations:** ^1^Professor in Pediatric Urology, Chairman of Urology Department in Imam Reza Hospital, Chairman of Regenerative Medicine Research Center (RMRC), Kermanshah University of Medical Sciences, Kermanshah, Iran; ^2^Professor in Pediatric Nephrology, Kermanshah University of Medical Sciences, Kermanshah, Iran; ^3^Urology Resident in Kermanshah University of Medical Sciences, Kermanshah, Iran

## Abstract

**Introduction:**

Vesicoureteral reflux (VUR) is a common urologic anomaly in children. Many techniques have been offered to manage this condition, in which one of them is modified Gil-Vernet antireflux surgery. The study fullfiled to evaluate the efficacy and safety of modified Gil-Vernet antireflux surgery in correction of high-grade VUR.

**Materials and Methods:**

A retrospective study in which we evaluated efficacy, safety, and complications of modified Gil-Vernet antireflux surgery as a choice procedure for high-grade reflux in all patients who underwent it since 2000 to 2016 at 2 hospitals of Kermanshah University of medical sciences that all of them were done by one surgeon.

**Results:**

183 patients with 290 high-grade refluxing units (grade IV or V) were reviewed. 182 refluxing units were grade IV, and 108 units were grade V. There were 76 (41.54%) patients with unilateral and 107 (58.46%) patients with bilateral VUR. Reflux in high-grade group corrected completely in 278 (95.86%) refluxing units and 175 patients (95.62%).

**Conclusions:**

Our results are remarkable and compatible with other techniques' results. This simple and safe technique can correct bilateral VURs simultaneously; thus, it is rational to be considered for high-grade VUR correction. According to our results, we suggest the modified Gil-Vernet antireflux procedure for high-grade VUR correction as a simple, safe, and successful technique. This trial is registered with 67145/86/1233.

## 1. Introduction

Vesicoureteral reflux (VUR) has been defined as nonphysiologic retrograde flow of urine from the bladder up the ureter into the kidney as the result of an insufficient vesicoureteral junction [1]. It is a common urologic anomaly in children. The prevalence of VUR is 0.4–1.8% of normal children and 30–50% among children with urinary tract infection [2, 3]. Untreated VUR can be associated with pyelonephritic scaring [4, 5]. The aims of management of a child with VUR are prevention of renal injury, recurrent febrile UTI, minimization of treatment morbidities, and follow-up duration [6]. Preferred therapy for a selected patient with VUR depends on different parameters as age, gender, VUR grade, symptoms, renal damage and function, laterality, bladder and bowel problems, compliance, and parents' choice [7]. Despite all of advancements, voiding cystourethrography (VCUG) is the gold standard of VUR which can demonstrate timing and laterality of the reflux, reflux grade, exact anatomy of the upper and lower urinary tracts, voiding phase, and anatomy of the bladder outlet and urethra [8]. High-grade VUR by IRS is defined as grade IV or V [1].

Treatment modalities include conservative (medical management and waiting for spontaneous resolution or downgrading) and interventional (open surgical, laparoscopic, or endoscopic approaches) [9]. Selecting surgical or medical management is of high controversy. Low-dose prophylactic antibiotic is the first line of treatment, and the cases of VUR should be permitted to resolve spontaneously [9]. However, in some conditions, surgical approach is inevitable. Multiple surgical procedures have been described to correct VUR. The surgeon can select the appropriate technique. The selective procedure is individualized according to the surgeon and patient condition [9]. The open surgical approaches can be classified as extravesical or intravesical based on the approach to the ureter. Gil-Vernet antireflux surgery is one of the intravesical approaches. Other conventional techniques such as Cohen and Leadbetter have high success rate, but stenosis of ureteral orifices, long time operation, long days of hospitalization, difficulty in catheterization, and malpositioning of ureteral orifices are their disadvantages. Gil-Vernet antireflux surgery is a useful and less invasive method that was introduced in 1983 and with fewer complications, bilaterally correction of VUR by one operation and prevention of reflux relapse in contralateral side which can occur in 10% of cases [10, 11].

It has many advantages such as shorter operating time and high success with lower complication rate; but in some literatures, this method has been accused of inefficacy in high-grade reflux [4]. In this study, the efficacy of Gil-Vernet antireflux technique in primary high-grade vesicoureteral reflux is assessed.

## 2. Methods

This study was a retrospective one in which we evaluated all the patients who underwent modified Gil-Vernet antireflux surgery as for their high-grade reflux from 2000 to 2016 in 2 hospitals of the Kermanshah University of Medical Sciences, and all of them were done by one surgeon. The study group was selected from patients with primary high-grade vesicoureteral reflux (grade IV or V) diagnosed by VCUG (X-ray contrast) with various complaints. The grading of vesicoureteral reflux was based on IRS radiologic classification of VCUG which was evaluated by a radiologist. *Indications for surgery were the failure of medical management, breakthrough UTI despite antibiotic prophylaxis, and the occurrence of new scars or worsening of renal function*. The exclusion criteria were relapsing reflux after other procedures or secondary reflux and patients with neurogenic bladder. The modified Gil-Vernet antireflux procedure was performed for all patients.

### 2.1. Surgical Technique

**Figure 1:**
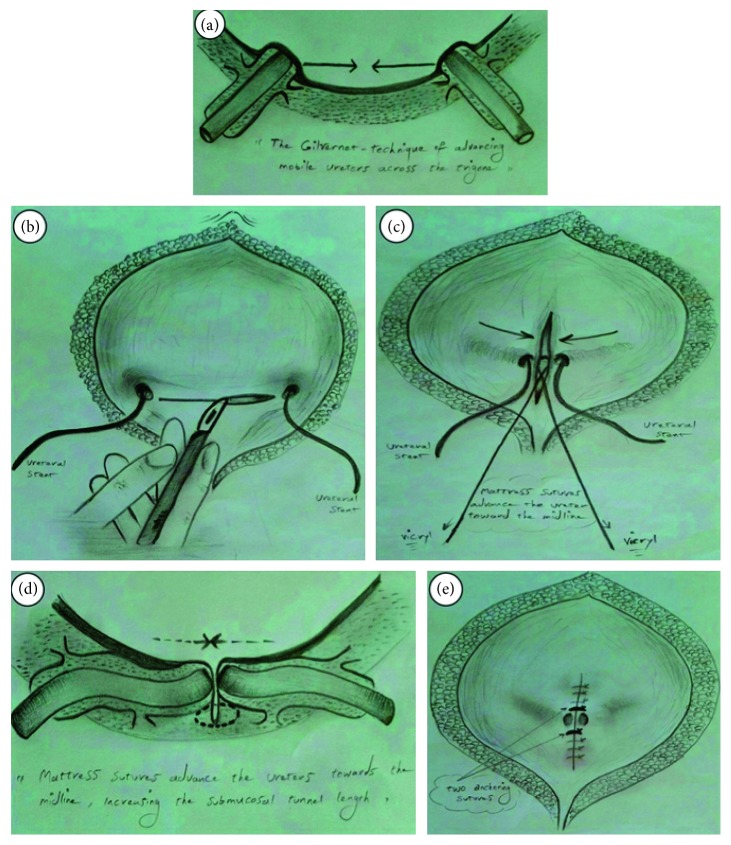
Schematic representation of the modified Gil-Vernet antireflux technique (drawn by Kaveh Kaseb).

A pfannenstiel skin incision was made. After splitting muscles and vertically opening the anterior bladder wall, two ureteral orifices were catheterized. A transverse incision is made through mucosa across superior aspect of trigone between ureteral orifices. The mucosa is then elevated off the bladder wall muscle carefully. Medial aspects of ureters gently dissected from surrounding tissues till hiatus and trigonal muscles up to advantis layer without damage to ureters in order to make the orifices free; in other words, waldeyer's sheath of bladder dissected completely from medial aspect of intramural ureter carefully so that by this action, approximation of ureter to midline would be tension free. It is an essential step to prevent lateral displacement of ureter orifice in future. One 5-0 vicryl suture was placed in a mattress fashion exactly near the orifice at the medial wall of each ureter to fix ureteral orifices in the midline, encompassing the periureteral sheath and muscle. This suture advances the ureters toward the midline tension freely, increasing their intramural length. The place of this suture is very important and must be on the edge of ureter's orifice, to prevent angulation of distal ureter near to the orifice. In addition to this, to prevent lateralization of ureters in future, 2 anchoring sutures were placed above and below the first suture. Mucosa repaired vertically with the 5-0 vicryl suture (Figure 1). Then, ureteral catheters were removed, and by intravenous injection of furosemide and observing the jet of urine from the ureteral orifices, their patency was confirmed. Surgery was ended by insertion and fixation of urethral catheter and repair of bladder water tight in two layers. No kind of drains was applied.

On the second postoperation day, if urine was clear, urethral catheter was removed and patient was discharged from the hospital. All patients visited one week after surgery and were followed by ultrasonography one month and radionuclide cystography (RNC) 3 months after surgery; if RNC was normal, follow-up imaging was done by using ultrasonography at 6 and 12 months after operation and then annually. If VUR was not resolved after 1 year, the patient was undergone reoperation by injection or different technique according to age, reflux grade, and the time passed from primary surgery. Also during annual follow-up sonography, if hydronephrosis or UTI was detected, the patient was evaluated by VCUG or RNC once again. Data of postoperative outcomes including days of hospitalization, reflux downgrading, complications, and the need of reoperation were collected and analyzed by SPSS-23.

## 3. Results

183 patients with 290 high-grade refluxing units were studied: 114 (68.3%) female and 69 (31.7%) male patients with mean age of 4.8 years (range 1–13 years). There were 76 (41.54%) patients with unilateral and 107 (58.46%) ones with bilateral VUR. 182 refluxing units were grade IV, and 108 were grade V. Mean operation time was 50 ± 10 minutes and mean hospitalization was 2.56 days (range 2–9 days). Mean follow-up time was 65 months (range 1–15 years). Reflux in the high-grade group had been corrected completely in 278 (95.86%) refluxing units and 175 patients (95.62%) (Table 1). The failure rate with persistent reflux was 8 patients (5 unilateral with grade IV VUR and 2 bilateral VUR with 1 side grade IV and other side grade V and 3 patients with unilateral grade V) with 12 refluxing units (4.1%). Six patients were retreated by ureteral submucosal injection of vantris which resolved finally. Two patients were managed by prophylactic antibiotic prescription. One postoperative complication occurred, and it was bladder dehiscence which at end repaired successfully. We had no complication, such as stenosis of ureteral orifices or ureteral obstruction, voiding dysfunction, contralateral VUR, urinary retention, and laboratory abnormalities.

## 4. Discussion

Vesicoureteral reflux is a common urologic anomaly in children. Untreated VUR can be associated with pyelonephritic scaring. Multiple surgical and medical procedures have been described to correct VUR. The basic steps of reimplantation are ureterolysis, creation of a new ureteral tunnel, the ureteral advancement, and anastomosis of the ureter with the bladder. In conventional open surgical techniques, despite high success rate, some complications are common and these procedures are invasive. The Gil-Vernet antireflux surgery has been accused of low efficacy in elimination reflux due to following reasons which are not effective especially in high-grade VUR [12]. And, in the latest edition of *Campbell-Walsh Textbook of Urology*, it has been cited “this procedure has been accomplished laparoscopically with limited success. Okamura and colleagues (1999) and Cartwright and colleagues (1996) reported success rates of 59% and 62.5%, respectively” [4]. Because of the relation between submucosal length and ureteral diameter in high-grade reflux, it is logical that the efficacy of Gil-Vernet operation may decrease [13]. The high success rate of other intravesical procedures has been cited in comparison to Gil-Vernet technique.

In Mirshemirani et al.'s study, 72 patients with VUR underwent Gil-Vernet antireflux surgery; success rate was 96.15%, and there was no postoperative complication [14]. Sharifi and Akhavizadegan reported that 39 women (18–65 years) underwent Gil-Vernet antireflux surgery with 97.95% success rate [15].

We believe modified Gil-Vernet antireflux surgery is simple with high success rate that has not gained attention and is a neglected operation in urology. Most conventional methods are more invasive, and many complications can occur. Contralateral VUR, urinary retention, voiding dysfunction, long operation time, long day's hospitalization, and obstruction are anticipated complications of different techniques of VUR repairment [4, 10, 15], that rarely occur in Gil-Vernet surgery. We had no complications such as mentioned one or ureteral obstruction; however, just in one case, we observed bladder dehiscence. Applying this modified technique resulted in high success rate, compatible with Gil-Vernet report [16]; however, Sharifiaghdas et al. have recently reported the lower success rate with long-term follow-up by trigonoplasty [17]. This technique is simple, safe, and least invasive. We had failure in 6 high grade refluxing units only. The relapse after operation occurred in patients with grade 4 VUR that managed with ureteral submucosal injection of vantris (promedon) and/or conservatively. In addition to all mentioned benefits of Gil-Vernet antireflux technique, other advantages of this technique are management of contralateral VUR without excessive intervention, and in cases of unilateral VUR, there is no need to follow-up contralateral side. This is the unique feature of Gil-Vernet antireflux technique. We believe as in high-grade VURs in which the orifices are lateralized, the maximum approximation of orifices is achieved by modified Gil-Vernet, and subsequently, more favorable results will be obtained. *Actually, it seems that more lateral ureter orifices more effective results for modified Gil-Vernet technique; thus as in high-grade VURs, lateral ectopia is more, and modified Gil-Vernet is more effective *. Our high success rate of modified Gil-Vernet antireflux surgery in contrast with previous studies results, may be, is due to the difference in our technique with classic Gil-Vernet antireflux surgery (the classic description by Gil-Vernet uses a single 3–0 nylon or prolene suture while we use 5-0 vicryl suture with 2 anchoring sutures higher and lower the cornerstone suture) and with more dissection of ureters till hiatus more submucosal ureteral length with approximation sheath technique of lateralized ureteral orifice is achieved.

In our personal experience, as we do and have done other antireflux techniques, it is obvious that “Gil-Vernet antireflux technique” has shorter operation time; however, though we emphasize the various advantages of the Gil-Vernet technique, as we did not compare different techniques related to their exact operation time, we cannot prove it statistically. In a recent study, Sarhan et al. have reported the necessity of surgical intervention in 89% of patient with primary high-grade VURs with success rate of 60% and 100% following endoscopic and surgical reimplantation, respectively [18].

Summarily, Gil-Vernet antireflux technique in contrast with Politano-Leadbetter technique is less invasive, with lower possibility of damage to adjacent organs and bilaterally operable simultaneously. Also in contrary to Cohen technique, there is no likelihood of ureteral angulation, and ureteral orifices are closer to their natural site which results in the easier retrograde catheterization and ureteroscopy in the future, and unlike the Glenn-Anderson technique, in the present technique, by less manipulation of trigone and less ureteral mobilization, the limitations of favorable submucosal ureter length creation are not present. Overall present technique is much easier than other one. Our experience indicates that the modified Gil-Vernet antireflux surgery is a very simple, safe, rapid, and highly successful procedure. We believe in its efficacy for high-grade VUR correction and thus suggest its application by other surgeon colleagues.

## Figures and Tables

**Table 1:** The basic statistics of the present study.

Gender	Side and grade				
	Left side and grade IV	Left side and grade V	Right side and grade IV	Right side and grade V	Bilateral high-grade
Female	18	10	16	7	63
Male	9	2	7	7	44
